# A universal coating strategy for inhibiting the growth of bacteria on materials surfaces

**DOI:** 10.3389/fchem.2022.1043353

**Published:** 2022-10-13

**Authors:** Jie Zhang, Min Wang, Liwei Hu, Qiang Zhang, Enni Chen, Zhongchao Wang, Yidong Shi, Lin Tan, Shimeng Xiao

**Affiliations:** ^1^ State Key Laboratory of Oral Diseases, Department of Periodontology, National Clinical Research Center for Oral Diseases, West China Hospital of Stomatology, Sichuan University, Chengdu, China; ^2^ College of Biomass Science and Engineering, Key Laboratory of Leather Chemistry and Engineering of Ministry of Education, State Key Laboratory of Polymer Materials Engineering, Sichuan University, Chengdu, China; ^3^ Yibin Institute of Industrial Technology/Sichuan University, Research Center for Fiber Science and Engineering Technology, Yibin, China; ^4^ Department of Periodontics & Oral Medicine, The Affiliated Hospital of Stomatology of Southwest Medical University, Luzhou, China

**Keywords:** dopamine-modified sodium alginate, polyhexamethylene guanidine, layerby-layer, universal antibacterial coating, functional cotton fabric

## Abstract

The development of a versatile antibacterial coating, irrespective of material characteristics, is greatly attractive but still a challenge. In this work, mussel-inspired dopamine-modified sodium alginate (SA-DA) was successfully synthesized as the adhesion layer, and antibacterial coatings on three types of substrates, namely cotton fabric, aluminum sheet, and polyurethane membrane, were constructed through the layer-by-layer (LbL) deposition of polyhexamethylene guanidine and sodium alginate. Among the coated materials, the coated cotton fabric was systematically characterized, and the results showed that it still exhibited ideal hydrophilicity, and its liquid absorption capacity increased with an increase in the coating layers. The growth of *Escherichia coli* and *Staphylococcus aureus* was notably inhibited on the coated cotton fabric, and 10 coating bilayers achieved 100% inhibition of bacterial growth within 10 min. Furthermore, an ideal antibacterial ability maintained after 10 cycles of antibacterial trials or 50 washing or soaping cycles. *In vitro* evaluation of the hemostatic effect indicated that the coated cotton fabric could promote blood clotting by concentrating the components of blood and activating the platelets, and no significant hemolysis and cytotoxicity were observed in the coated cotton fabric. Moreover, the coated aluminum and polyurethane film also displayed an obvious antibacterial effect, which proved that the constructed coating could successfully adhere to the metal and polymer surfaces. Therefore, this work provided a proper way for the progress of a current antibacterial coating tactics for different substrate surfaces.

## 1 Highlights

1. A universal coating strategy for inhibiting the growth of bacteria was constructed.
2. The durability and safety of layer-by-layer (LbL) coated surfaces was demonstrated.
3. Coated cotton fabric as a potential biomedical textile was systematically studied.
4. Versatile antibacterial coating is suitable for fabric, metal and polymer surfaces.

## 2 Introduction

Surface contamination by bacteria is ubiquitous, and almost all surface in everyday life and industrial production will be easily contaminated by bacteria (Klemm et al., 2010; Renner and Weibel, 2011). Bacteria can live on exposed surfaces for a few days or more, greatly increasing the risk of infectious diseases, the failure and infection of implants, and the corrosion damage to material and equipment facilities (Mahl and Sadler, 1975; Wissmann et al., 2021). For example, the overgrowth of bacteria on textile surfaces can lead to an unpleasant odor, dermatitis, and even other cross infections, while bacterial contamination on the surfaces of implants and wound dressings will pose a great threat to people’s life and health. In addition, the surface bacterial contamination of industrial equipment can lead to equipment failure, material corrosion, and cause economic losses (Monafo et al., 1976; Donlan, 2001; Hetrick and Schoenfisch, 2006; Procopio, 2022). Hence, surface bacterial contamination has become a significant threat to human health, industrial production safety, and even bringing a heavy burden on society. Obviously, fabricating antibacterial surfaces is one of the most prominent and effective ways to solve the issue of surface bacterial contamination, which generally first requires the formation of an active temporary surface by various surface modification approaches. Currently, many surface modification methods, such as chemical anchoring, host-guest interaction, and metal coordination interactions, have been employed to functionalize various surfaces (Mao et al., 2020; Wang et al., 2021; Yang et al., 2022). However, most surface coating methods are only specific to a certain type of surface or require specific pretreatment for the material substrates. Thus, there is a lack of general surface coating method applicable to different types of surfaces. As a surface binding protein secreted by mussels, biomimetic adhesive dopamine (DOPA) has been extensively studied and widely used as a universal adhesive compound for conjugating different materials onto a variety of substances, including fabrics, metals, and polymers (Ye et al., 2011; Dreyer et al., 2013; Jo et al., 2014; Kord Forooshani et al., 2019). More importantly, one of its most celebrated features is that the molecular structure of dopamine has characteristic reactive active sites, allowing for various secondary reactions (Tyo et al., 2019; Zhou et al., 2020). Therefore, multiple dopamine derivatives with the catechol moiety have been applied to surface modification.

Sodium alginate (SA), deriving from brown seaweeds, has widespread application in biomaterials for its nontoxicity, moderate production, biodegradability, economy, and biocompatibility (Varaprasad et al., 2020; Zhang and Zhao, 2020). However, pure SA normally performs insufficient adhesive capacity and poor mechanical properties for the structure of ¼ linked b-D-mannuronic acid (M) and C-5epimera-L-guluronic acid (G), thus its application in versatile coating was limited (Moskalewicz et al., 2021). Therefore, introducing dopamine groups into the molecular structure of SA through the amide reaction may endow it with an adhesive capacity. Polyhexamethylene guanidine (PHMG) is a kind of environment-friendly and efficient antibacterial agent with low toxicity in humans, offering a wide portfolio of applications in water treatment, wound disinfection and food packing (Fang et al., 2016; Cai et al., 2018; Ye et al., 2020; Zhang J. et al., 2021). It is worth noting that the immobilization of PHMG can enrich its application in the coating filed due to its excellent aqueous solubility as a coating material. Therefore, targeting the construction of a universal antibacterial coating system along with the combination of PHMG and SA via electrostatic interactions may endow various surfaces with desirable antibacterial properties with the assistance of dopamine groups, further enhancing the surface self-cleaning performance toward bacteria (Kord Forooshani et al., 2019).

**SCHEME 1:**
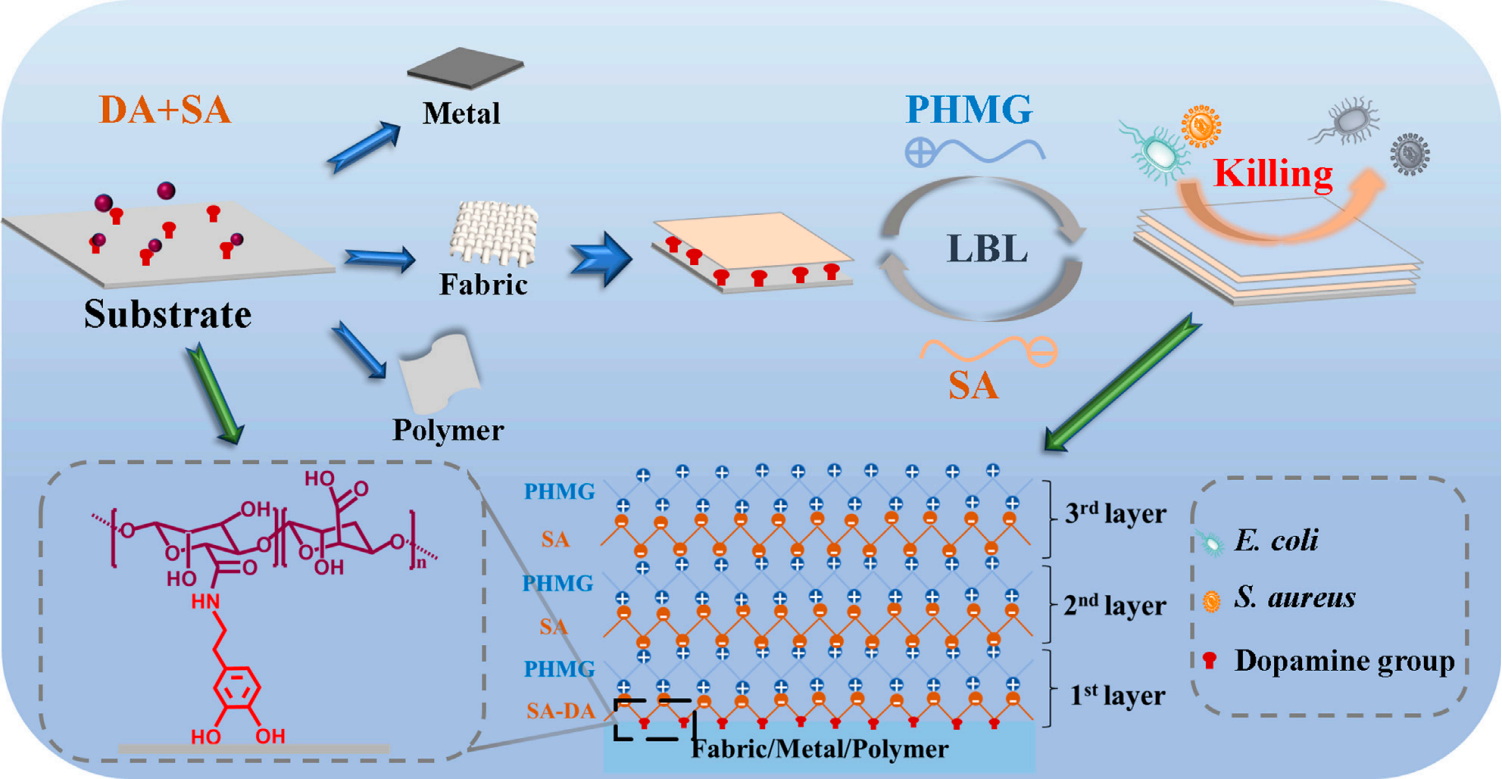
Schematic illustration for the preparation of the universal antibacterial coatings.

In this work, a biomimetic mussel adhesive protein was synthesized by modifying alginate with dopamine (SA-DA) via EDC/NHS chemistry, and the material was initially coated on the selected substrates as a result of the adhesion effect of dopamine. Afterward, the antibacterial coatings were constructed by means of the layer-by-layer (LbL) strategy on the above SA-DA layer surface via the successive alternating deposition of PHMG and SA (Scheme 1). This work aimed to build a manifold and multifunctional surface coating that was appropriate to different types of materials. To prove this design, we applied this coating method to a variety of substrates, including cotton fabric, aluminum sheet, and polyurethane membrane. Among the coated materials, the coated cotton fabric was systematically studied, including the basic properties of the cotton fabric, such as the cotton fabric microstructure, hydrophilicity, water absorption ability, water vapor transmission ability, antibacterial properties, and antibacterial stability. In addition, the hemostatic performance, cytocompatibility, hemocompatibility, and antibacterial ability were characterized and analyzed to explore the potential of its application as a wound dressing. Moreover, the desirable antibacterial properties of coated aluminum sheet, and polyurethane membrane were also demonstrated through the contact model. Therefore, we present a fresh universal coating and functionalization method in this work which would allow for the embellishment of antibacterial coatings on diversified surfaces and provide new impacts on a wide range of surface coatings and engineering applications.

## 3 Experimental section

### 3.1 Materials

Sodium alginate (SA), dopamine hydrochloride, 1-(3-dimethylaminopropyl)-3-ethylcarbodiimide hydrochloride) (EDC·HCl), and Tris-HCl buffer were provided by Chengdu Huaxia Chemical Reagent Co., Ltd., China. Polyhexamethylene guanidine hydrochloride (PHMG, 95%) came from Shanghai High Poly Biotechnology Co., Ltd. China. N-hydroxysuccinimide and 2-morpholinoethanesulphonic acid (MES) were supplied from Chengdu Kelong Chemical Co., Ltd., China. *Escherichia coli* (*E. coli*, ATCC8739) and *Staphylococcus aureus* (*S. aureus*, ATCC6538) were purchased by the R&D Lab of Functional Fibers at Sichuan University.

### 3.2 Synthesis of dopamine-modified sodium alginate (SA-DA)

The SA solution with a concentration of 1 wt% was prepared by dissolving the SA powder in deionized water until completely dissolved at room temperature. Afterward, EDC/NHS (where the monomer ratio was 1:1) was added to the solution to activate the carboxyl groups in SA. Next, MES was added, where its final concentration was set to 50 mM, and the blends was stirred for 30 min to make the solution homogeneous and stable. Then, the diluted hydrochloric acid (1 M) was employed to adjust the pH of the uniformly blended solution to pH 5.5. After 1 h, DA powder was added to the above SA solution, and the resulting solution was stirred for 12 h with the protection of N_2_ at room temperature. Finally, the product was precipitated three times with ethanol and then freeze-dried to obtain the dopamine-modified SA (SA-DA).

### 3.3 Structural characterization of SA-DA

After the SA-DA product was freeze-dried, the structure was characterized by FT-IR spectra (Tracer-100, Japan) and ^1^H NMR spectroscopy (NMR, Bruker AV III HD, 400 MHz, Germany). To further confirm the successful synthesis of SA-DA, an ultraviolet (UV)-vis spectrometer (UV2600A, China) was used to manifest the product, and the grafting ratio of dopamine was determined by setting a sequence of dopamine solutions with different concentrations as the standard.

### 3.4 Preparation of universal LbL antibacterial coatings

Cotton fabric, thermoplastic polyurethane (TPU) film and aluminum flake were used as substrate materials, the synthetic SA-DA was used as the adhesion layer, while the LbL coating was formed on the substrate surface by successively alternating the deposition of PHMG and SA at room temperature. The following were the operating steps. First, SA-DA was dissolved in deionized water to obtain a homogeneous solution (2 mg/ml), and tris-HCl buffer (pH = 8.5, 50 mM) was added. Then, the abovementioned clean substrates were immersed in the SA-DA solution and conditioned in a constant temperature shaker overnight to obtain the SA-DA coated substrates through dopamine adhesion. Next, the cleaned substrates were immersed in 2 mg/ml of PHMG for 10 min, and PHMG would deposit on the surface through electrostatic action. Then the substrates were washed with deionized water to detach the excess and dissociative PHMG on the surface and subsequently immersed in SA solution for 10 min. We repeated the above steps to obtain antibacterial coatings with different layers. Finally, the abovementioned coated substrates were cross-linked in a CaCl_2_ solution for 1 h and dried, and the coated cotton fabrics were selected for detailed research.

### 3.5 Surface characterizations of coated cotton fabric

The scanning electron microscopy (SEM, Hitachi SU3500, Japan) was employed to test the morphologies of the coated cotton fabric under the condition of 10 kV. A contact angle tester (Harke-SPCAX1, China) was brought to characterize the static water contact angle (WCA) of the coated cotton fabric using the sessile drop method, and at least five individual values were collected and averaged. The whiteness of each sample was measured for five times by a whiteness meter.

### 3.6 Interaction with liquid

#### 3.6.1 Liquid absorption rate

The liquid absorption rate of the coated cotton fabric was measured by means of the gravimetric method. The dried coated cotton fabrics (2 × 2 cm^2^) were immersed in deionized water for 12 h at 37°C, and then were brought out and weighed after removing any remanent water. The tests were run in triplicate and calculated by the following equation:

$$Liquid\;absorption\;rate=\frac{W_{t}-W_{0}}{W_{0}}\times 100\%,$$ (1)

where W_0_ denotes the dry weight of the samples, and W_t_ indicates the weight after wetting.

#### 3.6.2 Water vapor transmission rate

The water vapor transmission rate **(**WVTR) was demonstrated on the basis of the American Society for Testing and Materials (ASTM) E96-00 procedure. The coated cotton fabric was hermetically placed on the openings of a cylindrical glass bottle which contains 10 ml of deionized water. The water loss of the glass bottle was recorded respectively after 24 h under the humidity conditions of 35%, 55%, and 75% RH. WVTR was determined according to the weight loss (mg) by the functions of unit area (cm^2^) and time (h) at 37°C, and the glass bottles without samples were applied as comparisons. All the samples were tested three times, and the WVTR value was measured by the nether Equation 2:

$$\text{WVTR}\left(\text{mg}*{\text{cm}}^{2}*h^{-1}\right)=\frac{W}{S\times H},$$ (2)

where S indicates the exposure area (cm^2^), H represents the exposure time (h), and W is the weight (mg) change of the water.

### 3.7 Antibacterial assays

#### 3.7.1 Bactericidal assay

The antibacterial test of the coated cotton fabric against *E. coli* and *S. aureus* was performed in accordance with the modified AATCC 100 method. The process was conducted as follows. First, approximately 10^6^ CFU/ml of the bacterial suspensions were prepared with sterile phosphate-buffered saline (PBS) buffer as the bacterial strains were incubated to the logarithmic growth period. Afterwards, *E. coli* and *S. aureus* (25 μl) were added to the center region of the coated cotton fabric (2 × 2 cm^2^) and cultivated together at 37°C and 90% humidity for 10, 30, and 60 min. Next the samples were immersed in 1 ml of PBS buffer at setting intervals, and then 100 μl of eluent was equably spread on the LB agar plates and incubated at 37°C for 24 h. Subsequently, the antibacterial rate was calculated after counting according to the following formula:

$$I\text{nhibition}\;\text{rate}\left(\%\right)=\frac{Nc-N}{Nc}\times 100\%,$$ (3)

where N_C_ represents the number of colonies in the control groups and N indicates the experimental groups.

#### 3.7.2 Non-leaching demonstration test

The inhibition zone assay was resorted to comprehend the leaching status of the coated cotton fabric to certify that the passive antibacterial action of the samples completed through contact rather than the release of PHMG. Specifically, 100 μl of *E. coli* and *S. aureus* suspensions (∼10^6^ CFU/ml) were separately used to cover the full surfaces of the agar plates. Subsequently, the coated cotton fabrics before and after crosslinking were placed on the agar plates and cultured for 24 h at 37°C, and then they were observed for zones of inhibition formation.

#### 3.7.3 Bacterial morphologies observation

First, 10 ml of bacterial suspensions (*E. coli*, *S. aureus,* ∼10^8^ CFU/ml) were vortexed at 8,000 revolutions per minute and then mixed them thoroughly in 10 ml of PBS buffer. Then resuspended bacterial suspensions (5 ml) and coated fabrics (2 × 2 cm^2^) were co-cultured in a shaking water bath at 37°C for 12 h, and the untreated cotton fabrics co-cultured with bacterial suspensions were employed as a control. After that, the specimens were taken out and washed three times with 1 ml of PBS alternative. The bacteria were then adhered to the surface by immersing the specimens overnight at 4°C in an aqueous alternative containing 2.5% glutaraldehyde. Afterwards, the bacteria cells were dehydrated in a gradation of alcoholic solutions (30%, 50%, 70%, 90%, and 100%). The powders were then mixed with tert-butanol, freeze-dried, and finally examined by a SEM.

#### 3.7.4 Cyclic antibacterial test

The cyclic antibacterial capacity was determined through a similar method mentioned in Section 3.7.1. After each antibacterial test, the samples were desiccated at 60°C for the next cycle until 10 consecutive repeats were completed.

### 3.8 Antibacterial durability

The washing durability and soaping durability test were performed to evaluate the antibacterial durability of the coated cotton fabric according to the antibacterial fabric washing standard FZT 73023-2006. First, 3 L deionized water was added to the washing machine, and then the coated cotton fabric was added. Next, the fabric was washed for 25 min and drained. These steps were regarded as a cycle and counted as five washes, and these steps were repeated until 50 washes were completed. The soaping steps were similar to the washing steps, except 0.6 g of detergent was added during each washing process. In addition, a large amount of deionized water was required to completely remove the residual detergent on the fabric after the last soaping cycle, and then the cotton fabrics were dried. Subsequently, the antibacterial properties against *E. coli* and *S. aureus* of the samples were examined according to the description in Section 3.7.1.

### 3.9 *In vitro* blood coagulation test

Firstly, the prepared coated cotton fabric (circular shape, diameter of 1.5 cm) was placed on culture dishes at 37°C for 5 min. Afterward, each sample was dropped with 100 μl of whole blood (containing sodium citrate at a ratio of 1:9) and another 100 μl of whole blood was added to an empty culture dish served as a blank control. After 15 min of contact, the culture dishes were added with 25 ml of deionized water mildly without disturbing the clotted blood, and then the dishes were cultivated at 37°C with shaking at 30 rpm for 10 min. The experiment was repeated three times for each group. The solutions colors represented the level of blood coagulation, which was quantitatively determined by a UV spectrophotometer at 542 nm. Subsequently, the blood clotting index (BCI) of the samples was calculated by following equation:

$$BCI\;\left(\%\right)=I_{s}/I_{c}\times 100,$$ (4)

where I_s_ delegates the absorbance of the samples, and I_c_ suggests the absorbance of the blank control.

### 3.10 Coagulation mechanism investigation

In the first step, 20 ml of fresh, anticoagulant whole blood was spun at 1,500 revolutions per minute for 20 min. Because of the light-specific gravitation of the top plasma, the majority of platelets remained there. The top plasma was then extracted and spun as in the preceding stage to obtain a plasma rich in platelets that was over 70% pure. Afterwards, the samples (1 × 1 cm^2^) were maintained at 37°C for 1 h with 3 ml PRP. Afterward, all the samples were rinsed with PBS solution to remove the physically adhered platelets. The platelets were then fixed by soaking the above specimens for 2 h at room temperature within PBS with 2.5% glutaraldehyde. The platelets were then dehydrated for 15 min by immersing them in a succession of alcohol-PBS gradients (25%, 50%, 75%, 85%, 90%, 95%, and 100%). The specimens were then freeze-dried so that SEM could be employed to examine them.

### 3.11 Assessments of hemocompatibility

First, 10 min were spent spinning fresh rabbit blood at 3,500 rpm to isolate the red blood (RB) cells out from the supernatants. The supernatants were discarded. After that, the RB cells were cleaned and separated by centrifugation till the supernatant was completely clear. The RBCs were then suspended in PBS buffer at a 10% final concentration (v/v). At 37°C for 2 h, 1 cm^2^ of prepared cotton fabrics were inserted into a tube with 1 ml of RBC, and RBC suspensions diluted with PBS and DI water were employed as favorable and unfavorable controls, respectively. After allowing the whole blood alternatives remain for 2 h, they were vortexed at 3,500 rpm for 10 min. Then, every supernatant was gathered and a UV-Vis spectrophotometer was used to measure its absorbance at 540 nm to demonstrate that hemoglobin had been released. This testing was conducted 3 times, each time with specimens that were made by someone else. The following formula was utilized to find out the hemolytic activities:

$$\text{Hemolysis}\left(\%\right)=\frac{{\text{OD}}_{\text{sample}}-{\text{OD}}_{\text{negative}\;\text{control}}}{{\text{OD}}_{\text{Positive}\;\text{control}}-{\text{OD}}_{\text{negative}\;\text{control}}}\times 100,$$ (5)

where OD_sample_ represents the OD value of the experimental group, OD_positive_ indicates the OD value of the positive control group, and OD_negative_ expresses the OD value of the negative control group.

### 3.12 Cytocompatibility assessment

First of all, the coated cotton cloth was steeped for 24 h in the medium to acquire its extraction. Additionally, L929 cells were stored at 37°C with 5% CO2 for 24 h in tissue culture plates. Each well contained 5 × 10^3^ cells. Every well with cells was then loaded with the extraction and left to sit for 24 h. A positive control was the extraction of cotton fibers that had not been processed, while a negative control was the culture medium without extraction. At a fixed time, 200 μl of alternative CCK8 alternative was mixed into every well, and the culturing plates were left in the incubator for 2 h more. The absorbance at a frequency of 490 nm was then evaluated using a microplate reader. The equation below was utilized to find out how alive the cells were:

$$Cell\;viability\;\left(\%\right)=\left(A_{W}-A_{B}\right)/\left(A_{N}-A_{B}\right)\times 100,$$ (6)

Where A_W_, A_N_, and A_B_ represent the sample’s absorbance, the negative control’s absorption coefficient, and the blank control’s absorbance at 490 nm.

### 3.13 Evaluation of substrate versatility of the coatings

To evaluate the versatility of the coatings and demonstrate the successful formation of antibacterial self-assembly coatings on the aluminum and polyurethane surfaces, antibacterial tests were performed on coated aluminum and polyurethane according to the method described in Section 3.7.1.

### 3.14 Statistical analysis

All the experiments were measured three times at any rate. OriginPro9.1 software was devoted to analysis the statistic including calculating the average and mean ± standard deviation (SD).

## 4 Results and discussion

### 4.1 Chemical structure of SA-DA

**FIGURE 1:**
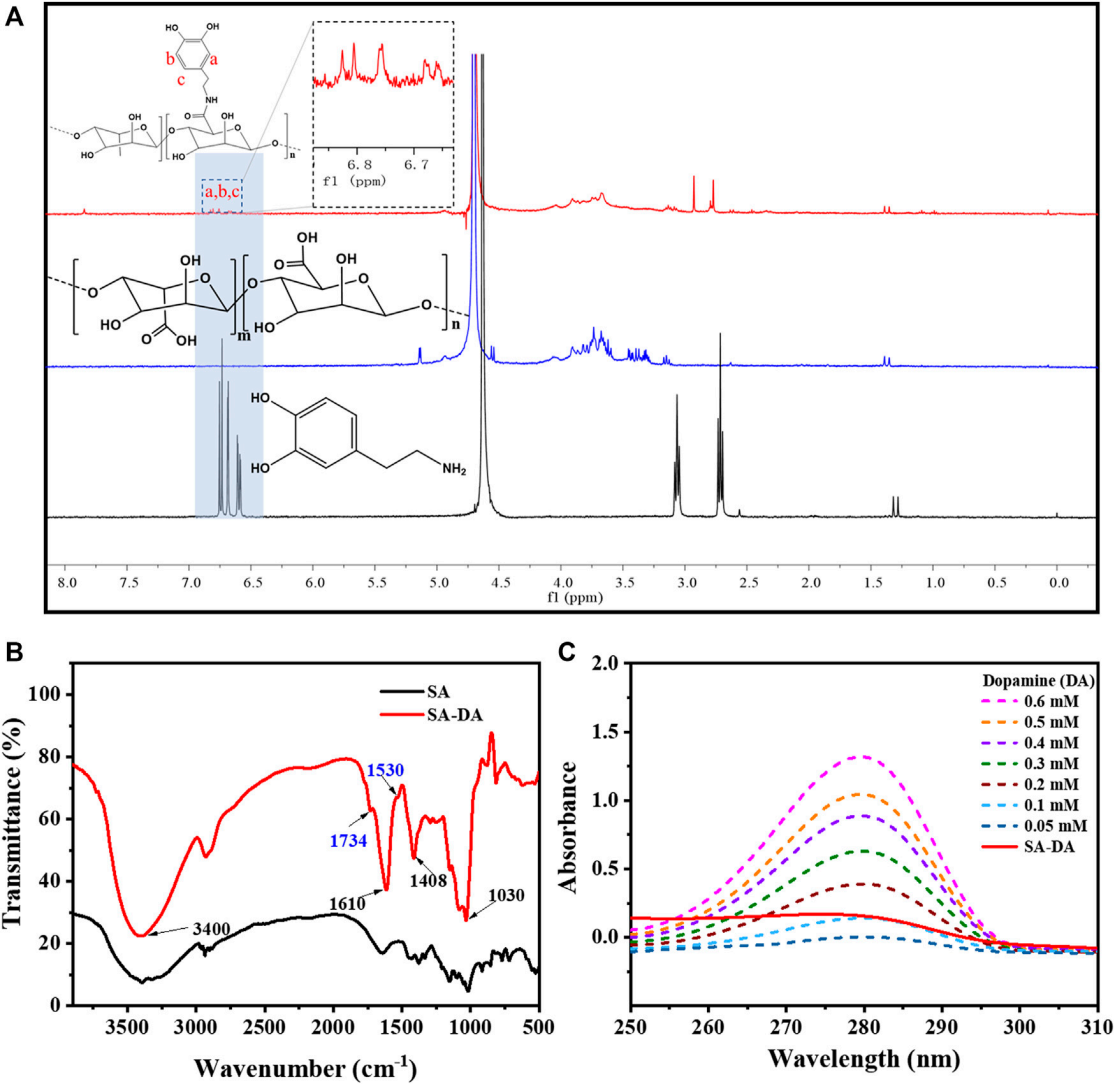
Characterization of SA-DA: **(A)** ^1^H NMR spectra of SA, DA, and SA-DA; **(B)** FT-IR spectra of SA and SA-DA; and **(C)** UV spectra of the dopamine and SA-DA solutions with different concentrations.

The structure characterization results for SA-DA are shown in Figure 1. According to the ^1^H NMR spectra (Figure 1A), ppm 6.6–7.0 was the H proton signal (a–c), which was connected to the aromatic proton in the dopamine structure (Zhang et al., 2020). Two wider peaks appeared at around ppm 3.2, which belonged to the proton signal of the methylene group close to the N (C=O) group (Hu et al., 2021). In addition, ppm 2.8 was the proton signal of the hydrogen on CH_2_CH_2_N (C=O) (Hu et al., 2021). Therefore, we inferred that the SA was successfully modified by dopamine.

Additionally, the FT-IR spectra (Figure 1B) further demonstrated the structure of the synthesized SA-DA. The wide peak at 3,400 cm^−1^ belonged to the stretching vibration peak of the hydroxyl group in the sample. The peaks that appeared at 1,610 and 1,408 cm^−1^ were attributed to the asymmetric and symmetrical contractions of the carboxyl group, respectively, while the peak near 1,030 cm^−1^ was the characteristic peak of C-O-C (Wang et al., 2013). Furthermore, the appearance of the fresh peak at 1,734 cm^−1^ was contributed by the stretching vibration peak of the carbonyl group in the amide structure, and the characteristic peak at 1,530 cm^−1^ was derived from the stretching vibration of -NH- in the amide structure (Wang et al., 2013). The analysis of the spectrogram demonstrated the reaction between the amine group of dopamine and the carboxyl group of SA was complete, and dopamine was successfully grafted onto SA.

The UV-Vis spectra of SA, DA, and SA-DA are shown in Figure 1A. Compared to SA, an obvious absorption peak was detected at 280 nm in the SA-DA sample, indicating the presence of the catechol group in the molecule, which again verified that dopamine was successfully grafted onto SA. In addition, the UV absorption of the dopamine solutions with different concentrations and SA-DA solutions are shown in Figure 1C. The grafting ratio was calculated as around 11.45% in line with the standard curve of dopamine (Figure 1B).

### 4.2 Properties of the coated cotton fabric

#### 4.2.1 Surface morphologies

**FIGURE 2:**
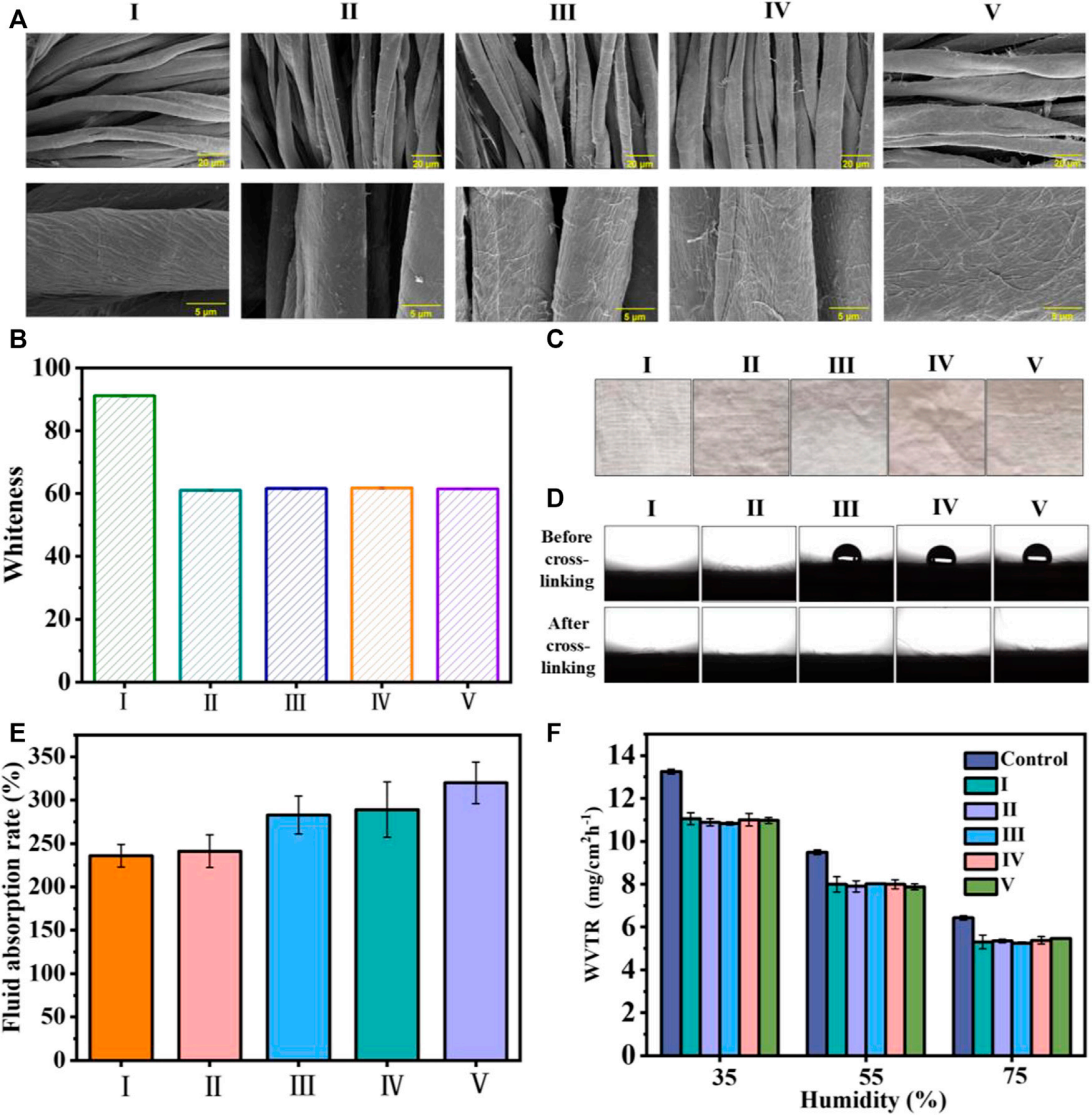
**(A)** SEM images of the cotton fabric with and without coating **(B)** the whiteness statistics of the cotton fabric before and after coating; **(C)** images of the cotton fabrics; **(D)** images and statistical results for WCA values; **(E)** liquid absorption rate of the uncoated cotton fabric and cotton fabrics with different coating layers; and **(F)** the WVTR of uncoated cotton fabric and coated cotton fabric under different humidity values. I) Cotton, II) cotton-SD, III) cotton-SD-PHMG_1_, IV) cotton-SD-PHMG_5_, V) cotton-SD-PHMG_10_.

The morphologies of the cotton and coated cotton fabrics are exhibited in Figure 2A. We observed that the cotton fabric fiber had natural distortion and texture on the surface, and the surface was relatively smooth. By contrast, the surface of the cotton fiber became rough after coating, and the surface roughness increased in compliance with the coating layers.

Typically, SA-DA solutions show a pale-yellow color but will become darker due to the oxidation of dopamine. Thus, the whiteness of the coated cotton fabric would decrease after coating. To quantitatively study the changes in the whiteness before and after coating, the whiteness values of the untreated cotton fabric and coated fabric were measured by a digital whiteness tester, and the results are shown in Figure 2B. Obviously, the whiteness value of the coated cotton fabric decreased significantly (from ∼91 to ∼60), and there was no significant difference in the whiteness value of the cotton fabric cloth with different coating layers, indicating that the number of coating layers had no obvious effect on the whiteness value. In addition, as shown in Figure 2C, the color of the cotton fabric after coating became significantly darker, which was consistent with the whiteness value test results. In addition, the color of the cotton fabric after coating became significantly darker in Figure 2C, which was consistent with the whiteness value results.

The surface wettability of the cotton fabric with and without the coating is summarized in Figure 2D, where all the samples exhibited excellent hydrophilic properties, and the little water drops were absorbed immediately as they get to the sample surfaces. Additionally, the contrast experiments between cross-linked and uncross-linked cotton fabrics were carried out to explain why cotton-SA-PHMG_1_, cotton-SA-PHMG_5_, and cotton-SA-PHMG_10_ maintained great hydrophilicity. The photographs in Figure 2D demonstrated that the hydrophilicity of the coated cotton fabric increased significantly after cross-linking with CaCl_2_, and the WCA decreased to 0°. The above phenomenon could be explained by CaCl_2_ cross-linking, which changed the arrangement direction of the PHMG hydrophobic long chain (Azeredo et al., 2012). Therefore, CaCl_2_ cross-linking could improve the hydrophilicity of the coated cotton fabric.

### 4.3 Liquid absorption rate and WVTR

According to Figure 2E, the liquid absorption ability of the coated cotton fabric enhanced obviously as increasing the coating layers, and the reason rooted in that SA could absorb more liquid after forming a coating on the surface. In addition, the WVTR of the uncoated and coated cotton fabrics was recorded, as demonstrated in Figure 2F. We found that the WVTR values of the samples displayed a downward trend with an increase in humidity, as higher humidity was not conducive to water vapor evaporation. In addition, under the same humidity, the WVTR of the cotton fabric before and after coating exhibited tiny change, which indicated that the coating did not affect the WVTR of the cotton fabric.

### 4.4 Antibacterial properties

#### 4.4.1 Bactericidal efficiency

**FIGURE 3:**
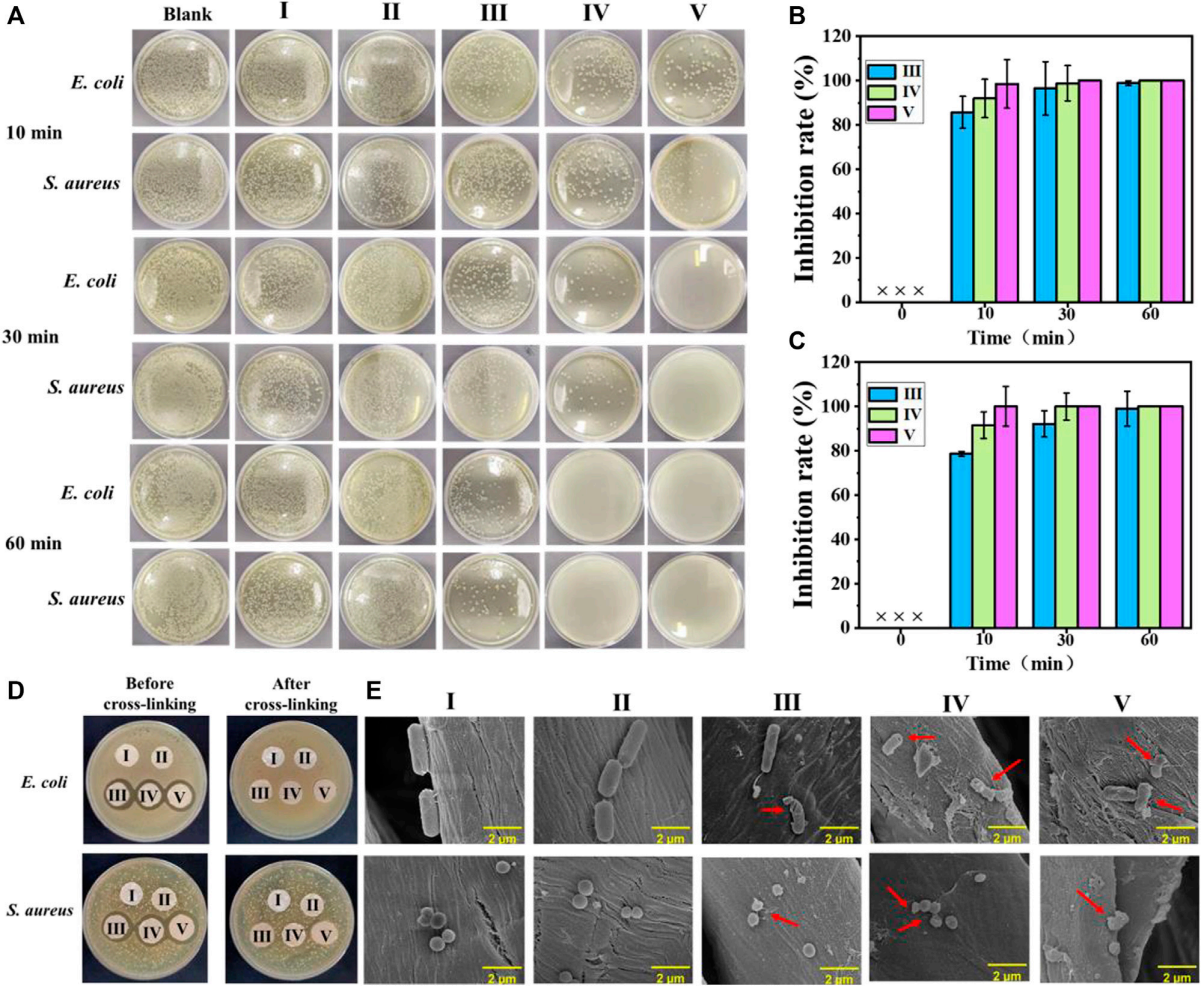
**(A)** Photographs showing antimicrobial activities of coated cotton fabrics against *E. coli* and *S. aureus* after different contact times; the sterilization rate histogram of the coated cotton fabric against *E. coli* **(B)** and *S. aureus* **(C)**; **(D)** inhibition zone of the coated cotton fabric before and after cross-linking against *E. coli* and *S. aureus*; and SEM images of *E. coli* and *S. aureus* in the uncoated and coated cotton fabric groups **(E)**. I) Cotton, II) cotton-SD, III) cotton-SD-PHMG_1_, IV) cotton-SD-PHMG_5_, V): cotton-SD-PHMG_10_.

Normally, the untreated cotton fabric will be easily colonized by a diverse range of microorganisms. Figure 3A shows the bactericidal activities of the experimental samples against *E. coli* and *S. aureus*, where the bactericidal rate histogram of cotton-SA-PHMG_1_, cotton-SA-PHMG_5_, and cotton-SA-PHMG_10_ are displayed in Figure 3B (against *E. coli*) and 3C (against *S. aureus*). Clearly, cotton-SA-PHMG_10_ had the best antibacterial performance, where it killed 98.54% of *E. coli* and 99.98% of *S. aureus* within 10 min, and nearly 100% of both bacteria were inactivated within 30 min. Additionally, ideal antibacterial efficiency was demonstrated in the cotton-SA-PHMG_1_ and cotton-SA-PHMG_5_ groups for killing all bacteria within 1 h. These results indicated that the sterilization rate of the coated cotton fabric was closely related to the contact time and the number of coating layers, where the longer the contact time (sufficient interaction with bacteria) and the greater the number of coating layers (sufficient antibacterial active sites), the higher the sterilization rate.

#### 4.4.2 Observation of the inhibition zone

An inhibition zone assay was adopted to investigate the leaching characteristics of the coated cotton fabrics before and after cross-linking. The results are shown in Figure 3D. An obvious inhibition zone would appear as the antibacterial performance was completed by the leaching of PHMG and the inhibition zone would become larger with an increasing amount of PHMG. Obviously, no visible inhibition zones were observed in the series of uncoated and coated cotton fabrics after cross-linking, where the reason for the former was attributed to the weak antibacterial activity, and the explanation for the latter was on account of no obvious leaching of antibacterial substances. However, the coated cotton fabric without crosslinking showed an obvious inhibition zone, with a greater number of coating layers and a larger inhibition zone, which was caused by the leaching of PHMG in the coating. Therefore, it was necessary to reduce the leaching of PHMG through cross-linking, as it could avoid potential harm to the human body and the environment, as well as offer the benefit of long-term antibacterial capacity. In other words, the coated cotton fabrics after cross-linking with CaCl_2_ performed better safety than not cross-linked.

#### 4.4.3 Bacterial morphology

As the SEM imagines show in Figure 3E, the morphologies of the *S. aureus* treated with the untreated cotton fabric and SA-DA coated cotton fabric were rod-shaped while the *E. coli* were spherical. However, after incubating with cotton-SA-PHMG_1_, cotton-SA-PHMG_5_, and cotton-SA-PHMG_10_, the bacterial morphology appeared to undergo deformation or disruption as the arrows pointed out in the SEM imagines. The variations resulted from the positively charged domain and hydrophobic alkyl groups of PHMG for functioning on the bacterial cell wall and membrane which was negatively charged and finally leading to cell lysis (Chen et al., 2020; Zhang J. et al., 2021; Zhou et al., 2022).

### 4.5 Antibacterial stability

#### 4.5.1 Cyclic antibacterial capacity

**FIGURE 4:**
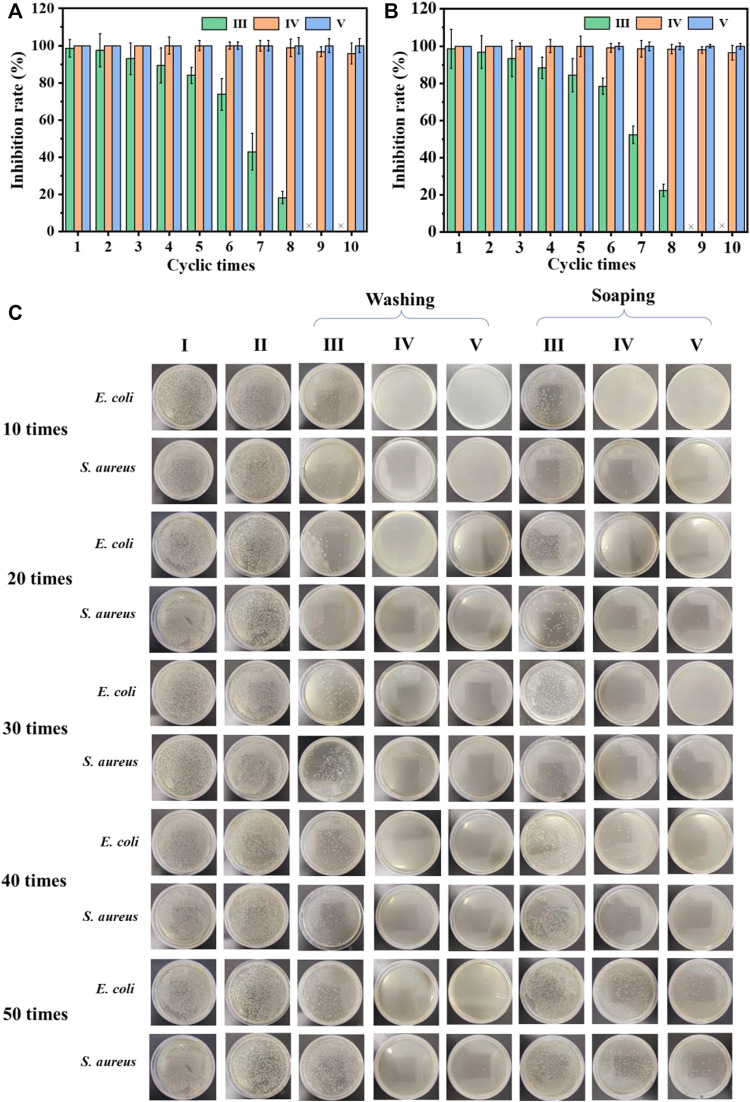
The recycling antibacterial results of the uncoated cotton fabric and coated cotton fabric against *E. coli* **(A)** and *S. aureus* **(B)**; the bactericidal activity of the coated cotton fabric against *E. coli* and *S. aureus* after different washing and soaping cycles **(C)**. I) Cotton, II) cotton-SD, III) cotton-SD-PHMG_1_, IV) cotton-SD-PHMG_5_, V) cotton-SD-PHMG_10_.

Considering the repeated use of the cotton fabric in daily life, antibacterial recycling tests were performed. As shown in Figures 4A,B, after eight cycles of antibacterial experiments, the cotton-SD-PHMG_1_ had completely lost its antibacterial ability. For the cotton-SD-PHMG_5_, its sterilization rate still reached more than 98%. Notably, the sterilization rate of cotton fabric-SD-PHMG_10_ against *E. coli* and *S. aureus* could still maintain more than 99.9% even after 10 antibacterial cycles. As a large number of bacteria would contact with the fabrics during the antibacterial experiment, thus bacterial debris inevitably and gradually accumulated on the surface of testing fabrics accompanied by repeated antibacterial cycles, thus leading to the decline of antibacterial ability (Zhang et al., 2022).

#### 4.5.2 Antibacterial durability

The antibacterial durability of the cross-linked coated cotton fabric was measured by means of washing and soaping experiments. The antibacterial effect of cotton-SD-PHMG_1_ decreased significantly after 30 washing cycles according to Figure 4C, while only 20 soaping cycles depressed its antibacterial activity (the fabric was washed for 25 min and drained). For the cotton-SD-PHMG_5_, it could still maintain an ideal antibacterial effect even after washing 50 times, while its antibacterial ability descended significantly after 50 soaping cycles. Of note, the cotton-SD-PHMG_10_ maintained an excellent antibacterial effect even after 50 washing or soaping cycles. Therefore, as the number of coating layers grew, the washing resistance of the coated cotton fabric became more excellent. Furthermore, the washing resistance of the coating was better than the soaping resistance. Therefore, cotton-SD-PHMG_10_ had the potential to be a reusable antibacterial textile.

### 4.6 *In vitro* biological properties

#### 4.6.1 Blood coagulation function

**FIGURE 5:**
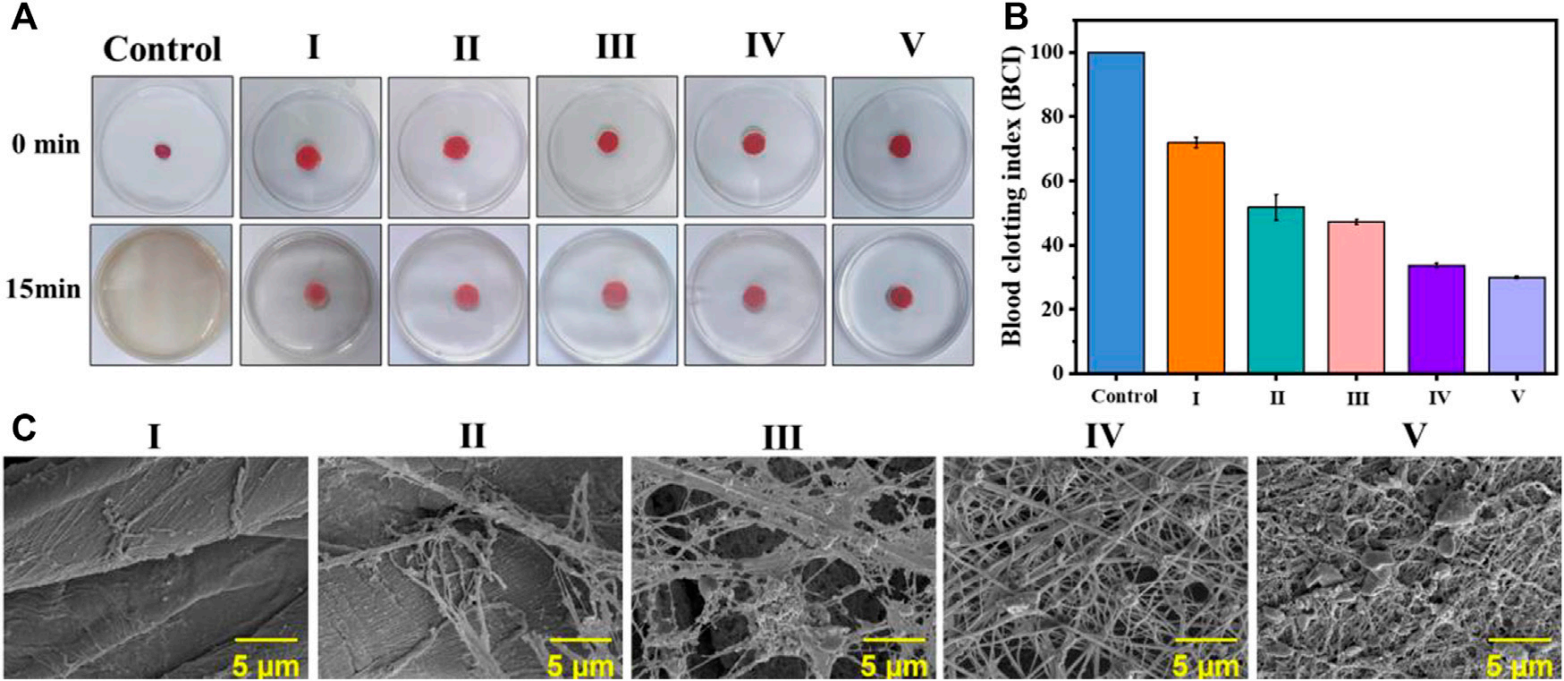
*In vitro* blood coagulation and hemolysis assessment on the coated cotton fabric:**(A)** the photographs of the blood-clotting process; **(B)** the BCI value of whole blood clotting evaluation of the coated cotton fabric, nonwoven and control; and **(C)** platelet pseudopods and fibrin network formed on different samples. I) cotton, II) cotton-SD, III) cotton-SD-PHMG_1_, IV) cotton-SD-PHMG_5_, V) cotton-SD-PHMG_10_.

As a type of traditional medical dressing, cotton fabric lacks antibacterial and coagulation functionality. To explore the feasibility of coated cotton fabric as an antibacterial and hemostatic dressing, an *in vitro* coagulation test was performed as Figure 5A. The BCI value of the coated cotton fabric group was dramatically lower than the blank control group as demonstrated in Figure 5B. Moreover, the coagulation effect of the coated cotton fabric was enhanced as the number of self-assembled layers increased, indicating that the coating on the surface of the cotton fabric was conducive to clotting.

#### 4.6.2 Hemostatic mechanism

As the platelets contribute to physiological hemostasis and pathological thrombo-inflammation primarily, platelet adhesion and morphology are significant elements for assessing the hemostatic properties of materials. Hence, to further investigate the interaction between coated cotton fabric and platelets, SEM analysis was performed after incubating the cotton fabric with the PRP before and after coating. As shown in Figure 5C, a dense fibrin network formed on the surface of the coated cotton fabric after contact with the PRP, where the more coating layers, the denser the fibrin network. However, it did not appear in the cotton fabric and SA-cotton fabric groups, which further proved the coagulation-promoting effect of the coating. This confirmed that the platelets on the surface of the cotton-SA-PHMG were activated, which was attributed to the effect of PHMG. The guanidine groups of PHMG are electropositive which have interactions with electronegative cell membranes on the red blood, leading to charming hemagglutination and adsorption of fibrinogen and plasma proteins, as well as accelerate the aggregation of the platelets (Gharibi et al., 2019; Zhang J. et al., 2021; Zhang et al., 2022). Additionally, the carboxyl groups on SA came into being the complex with the Fe^3+^ ions which were escaped from the damaged RBCs, resulting in the aggregation and adhesion of platelets (Sekhon and Sen Gupta, 2018; Zheng et al., 2021). Therefore, the coated cotton fabric could be used as an antibacterial and hemostatic dressings.

### 4.7 Hemocompatibility

**FIGURE 6:**
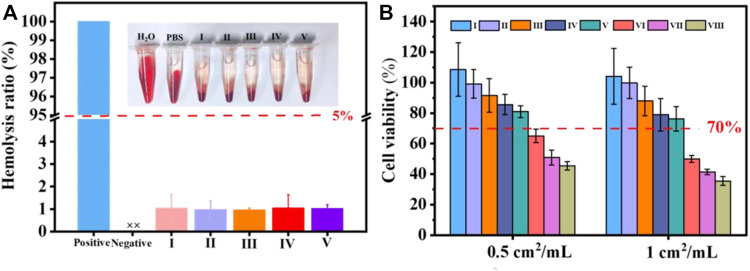
**(A)** Hemolysis of coated cotton fabric against erythrocyte cells; **(B)** cell viability with or without coated cotton fabric extracts treatments. I) cotton, II) cotton-SD, III) cotton-SD-PHMG_1_, IV) cotton-SD-PHMG_5_, V) cotton-SD-PHMG_10_, VI) uncrosslinked-cotton-SD-PHMG_1_, VII) uncrosslinked-cotton-SD-PHMG_5_, VIII) uncrosslinked-cotton-SD-PHMG_10_.

To demonstrate the security of the coated cotton fabric as a wound covering, a hemolytic test was conducted. As exhibited in Figure 6A, the hemolysis rate of the cotton fabric before and after coating showed unapparent difference. According to the ASTM F756-08 standard, cotton-SD-PHMG_10_ played inappreciable hemolytic activity for exhibiting a hemolytic rate of 1.02 ± 0.17%, which was much lower than 5%. As a result, the defects caused by hemolysis, such as an undesired immune response, the formation of thrombosis, and inflammation at the wound site, can be avoided.

### 4.8 Cytocompatibility

To further evaluate the safety of the prepared coated cotton fabric on the human body, a cytotoxicity experiment was conducted. According to Figure 6B, the cell viability decreased as the number of coating layers increased, and the direct reason derived from that more PHMG leached from the cotton fabrics which resulted in weaker cytocompatibility. In addition, comparing to the cytotoxicity of the coated cotton fabric before and after cross-linking, the cytotoxicity of the coated cotton fabric after cross-linking was greatly reduced. This was because cross-linking limited the leaching of PHMG, which was consistent with the inhibition zone experiment results. The cell viability of the uncross-linked coated cotton fabric group was recorded even below 50%, while the leaching liquid assay exhibited that more than 80% of cells were viable at extract ratios of 0.5 cm^2^/ml and surpassing 70% at extract ratios of 1 cm^2^/ml. According to the International Standard Organization (ISO/EN 10993-5) recommendations, the testing materials are cytotoxic as the cell survival rates are lower than 70%. Therefore, the cross-linked coated cotton fabric was relatively safe and innocuous to the human body.

### 4.9 Versatility of the coating

**FIGURE 7:**
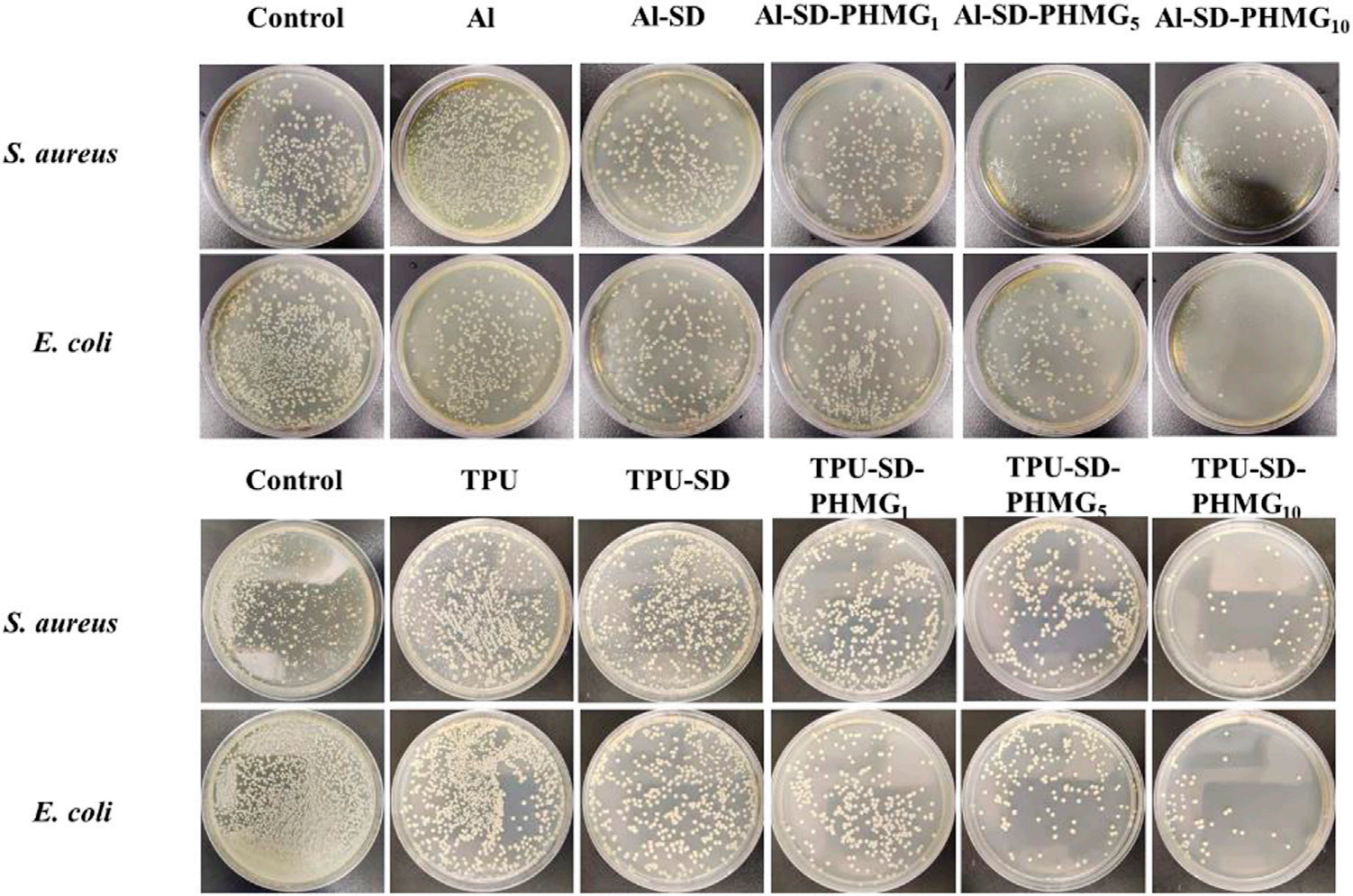
Antibacterial results of the coated Als and coated TPU.

To verify the versatility of the coating, the antibacterial performance of the coated aluminum sheet and TPU membrane was preliminarily investigated. As shown in Figure 7, the untreated (Al and TPU) and SA-DA only treated (Al-SD and TPU-SD) aluminum sheet and TPU membrane showed no antibacterial effect. Both Al-SA-PHMG_1_ and TPU-SA-PHMG_1_ showed a slight antibacterial effect, which gradually strengthened with an increase in the number of coating layers, indicating the successful formation of the SA and PHMG self-assembled coatings on their surfaces. Therefore, we concluded that the coating not only formed on the surface of the cotton fabric but also on the metal and polymers. However, the antibacterial effect of the coated metal and polymer membrane was weaker compared to that of the coated cotton fabric, which indicated that the coating was easier to formed on the cotton fabric. The possible reasons can be attributed to those that the surface of cotton fabric is rougher and has more active groups, which are conducive to the adhesion of dopamine groups, thus making it easier to form the coating on its surface.

## 5 Conclusion

Dopamine was successfully grafted on SA, and a versatile antibacterial coating was successfully obtained on various surfaces, including cotton fabric, aluminum sheet, and TPU membrane, via the LbL strategy. The produced antibacterial coated cotton fabric showed hydrophilic characteristics, admirable liquid absorption ability, and an excellent WVTR and sterilization ability. Additionally, the coated cotton fabric showed outstanding antibacterial stability and antibacterial durability. Furthermore, the coated cotton fabric showed a remarkable coagulation ability, desirable cytocompatibility, and hemocompatibility. Notably, the coated aluminum sheets and TPU membrane also showed ideal antibacterial properties with increasing the coating layers, which proved that the coating and the corresponding method was generally universal. Therefore, the coating material and modification approach developed in this investigation exhibit the potential to multifarious materials for inhibiting the growth of bacteria on surfaces.

## Data Availability

The original contributions presented in the study are included in the article/Supplementary Material, further inquiries can be directed to the correspnding author.
