# Supplementary figures and images for: A universal coating strategy for inhibiting the growth of bacteria on materials surfaces
Source: Front Chem. 2022 Oct 13;10:1043353. doi: 10.3389/fchem.2022.1043353 (PMC9606354; doi:10.3389/fchem.2022.1043353)

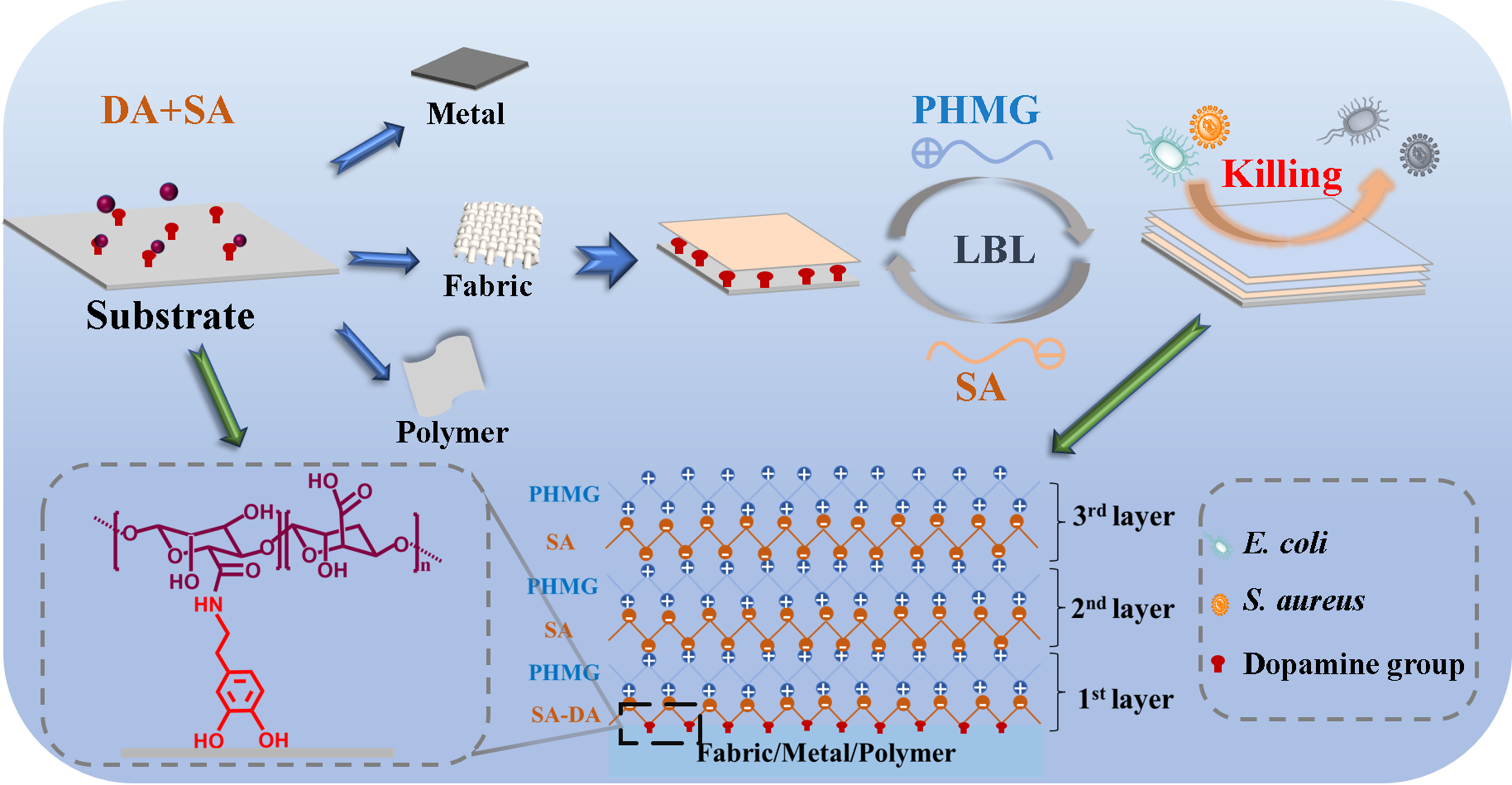

Supplement: Supplementary file 1 [file Image1.TIF]
