# Supplementary material for: A universal coating strategy for inhibiting the growth of bacteria on materials surfaces
Source: Front Chem. 2022 Oct 13;10:1043353. doi: 10.3389/fchem.2022.1043353 (PMC9606354; doi:10.3389/fchem.2022.1043353)
Supplement: Supplementary file 2 [file DataSheet1.docx]

Supporting Information

**A universal coating strategy for inhibiting the growth of bacteria on materials surfaces**

Jie Zhang^1,2,3#^, Min Wang^2,3#^, Liwei Hu^1^, Qiang Zhang^2^, Enni Chen^1^, Zhongchao Wang^4^, Yidong Shi^2,3^, Lin Tan^2,3^, Shimeng Xiao^1*^

*^1^State Key Laboratory of Oral Diseases, Department of Periodontology, National Clinical Research Center for Oral Diseases, West China Hospital of Stomatology, Sichuan University, Chengdu, 610041, China.*

*^2^College of Biomass Science and Engineering, Key Laboratory of Leather Chemistry and Engineering of Ministry of Education, State Key Laboratory of Polymer Materials Engineering, Sichuan University, Chengdu 610065, China.*

*^3^Yibin Institute of Industrial Technology/Sichuan University, Research Center for Fiber Science and Engineering Technology, Yibin Park, Yibin 64460, China.*

*^4^Department of Periodontics & Oral Medicine, The Affiliated Hospital of Stomatology of Southwest Medical University, Luzhou 646000, China.*

*Corresponding to: Dr. Shimeng Xiao (E-mail:* *[shimengxiao817@163.com](mailto:XXXX@scu.edu.cn))*

*^#^These two authors contributed equally to this work.*

1. UV analysis

In order to further confirm the successful synthesis of SA-DA, the product was characterized by a UV spectrophotometer. Firstly, the UV baseline scanning was performed with deionized water, and then SA, DA and SA-DA were dissolved in deionized water respectively for full-wavelength scanning, and thus to obtain the full-wavelength curves of SA, DA and SA-DA. In addition, the standard curve of DA was drawn according to the absorbance of DA with different concentrations at the maximum absorption wavelength.

|   (a) |   (b) |
| --- | --- |

**Fig. S1** The full wavelength curves of SA, DA and SA-DA (a); the standard curve of DA (b).

2. Observation of the inhibition zone

The inhibition zone assay was conducted to demonstrate the passive antibacterial action of the coated cotton fabric completed through contact rather than the release of PHMG. Specifically, 100 μL of *E. coli* and *S. aureus* suspensions (~10^6^ CFU/mL) were separately used to completely cover the surfaces of the agar plates. Subsequently, the coated cotton fabrics before and after crosslinking were placed on the agar plates and cultured for 24 h at 37 °C, then the inhibition zones were observed, and the pictures after removing the fabrics were also recorded. Obviously, inhibition zones derived from the non-crosslinked fabrics are larger those from the crosslinked ones, and no bacterial colonies appeared below all the fabrics (**Fig. S2a**), and the possible reason was attributed to that the underlying bacteria were absorbed by the cotton fabrics. In order to verify the speculation, we further rinsed the cotton and cotton-SD-PHMG5 with PBS buffer, and 100 μL of each eluant was used to spread on the LB agar plates and incubated at 37 °C for 24 h. **Fig. S2b** demonstrated that alive bacteria existed on the blank cotton, while no alive bacteria existed on the cross-linked cotton fabric or non-crosslinked one. Therefore, the inhibition zone can only be observed visually upon the leaching of PHMG, in other words, PHMG was stabilized on the coated cotton fabric through crosslinking.


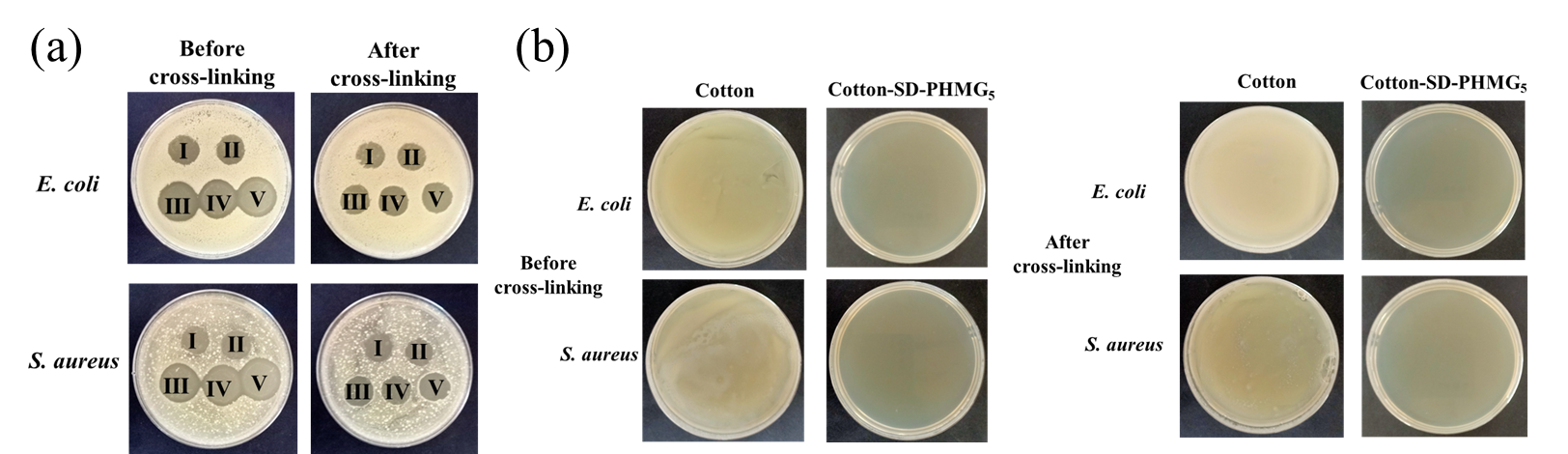


**Fig. S2** (a) Inhibition zone after removing the coated cotton fabric before and after cross-linking; (b) antibacterial test against *E. coli* and *S. aureus.*
